# Thoracoscopic repair of esophageal atresia with distal tracheoesophageal fistula: is it a safe procedure in infants weighing less than 2000 g?

**DOI:** 10.1007/s00464-020-07538-z

**Published:** 2020-04-22

**Authors:** Joonhyuk Son, Yerang Jang, Wontae Kim, Sanghoon Lee, Ji Seon Jeong, Suk-Koo Lee, Jeong-Meen Seo

**Affiliations:** 1 Department of Surgery, Samsung Medical Center, Sungkyunkwan University School of Medicine, 81 Irwon-Ro, Gangnam-Gu, Seoul, 06351 Korea; 2 Department of Anesthesiology, Samsung Medical Center, Sungkyunkwan University School of Medicine, Seoul, Korea

**Keywords:** Esophageal atresia, Tracheoesophageal fistula, Thoracoscopy, Minimally invasive, Low birth weight

## Abstract

**Background:**

Since Rothenberg first performed thoracoscopic repair for esophageal atresia with distal tracheoesophageal fistula (EA/TEF) successfully in 2000, thoracoscopic repair has achieved status as a routine procedure worldwide. Previously, an international multicenter study reported that this procedure was not inferior to conventional open surgery. However, thoracoscopic surgery is a highly difficult operation for surgeons and anesthesiologists; as a result, the safety and efficacy of the surgery is still under debate. Considering these circumstances, the purpose of this study was to analyze the results of single-center thoracoscopic surgery and to compare the outcomes relative to the patient’s weight at the time of surgery.

**Methods:**

We retrospectively analyzed patients with EA/TEF who underwent thoracoscopic surgery in a single center between October 2008 and February 2017.

**Results:**

In total, 41 cases of thoracoscopic repair of EA/TEF were performed. Upon subgrouping by over and under 2000 g of body weight at the time of operation, 34 were found to be over 2000 g and seven were under 2000 g. Intraoperative factors and events were not significantly different between the two groups. Additionally, most of the postoperative outcomes, including the rate of postoperative leakage and strictures, showed no difference. On the other hand, the under 2000 g group had more gastroesophageal reflux requiring fundoplication than did the heavier group (*P* = 0.04).

**Conclusions:**

The results of this center’s thoracoscopic repair of EA/TEF were not inferior to other centers’ outcomes. Additionally, the intraoperative and postoperative outcomes were similar despite differences in weight at operation. Therefore, thoracoscopic repair might be a feasible surgical option for infants weighing less than 2000 g when performed by a surgeon and anesthesiologist team who are experienced in pediatric thoracoscopic surgery.

Minimally invasive surgery for esophageal atresia with distal tracheoesophageal fistula (EA/TEF) had garnered increasing interest among pediatric surgeons since Rothenberg first successfully performed thoracoscopic repair in an infant with EA/TEF [1]. As a result, in 2005, an international multicenter study with 104 cases reported the results of thoracoscopic repair for EA/TEF [2]. Comparing the technique with conventional open surgery, the study demonstrated better results for the thoracoscopic surgery except for stenosis requiring balloon dilatation [3–7]. However, thoracoscopic repair is a technically challenging procedure for many pediatric surgeons to perform. Moreover, EA/TEF includes numerous intraoperative critical events that require an anesthesiologist’s intervention; as a result, the presence of a skillful anesthesiologist is also needed [8]. For these reasons, contraindications for thoracoscopic repair of EA/TEF have been proposed, such as cardiorespiratory instability and birth weight under 2000 g [9]. Thus, the safety and efficacy of the thoracoscopic procedure remain under debate. Considering these circumstances, the purposes of this study were to analyze the results of single-center thoracoscopic surgery and to compare the outcomes relative to body weight at the time of surgery.

## Materials and methods

Since the first case of thoracoscopic repair of EA/TEF was performed in October 2008, this procedure has been considered as the surgery of choice in our center for patients who required EA/TEF repair (Samsung Medical Center, Seoul, Korea) [10]. As our experience grew, a number of thoracoscopic cases also have increased gradually over the years. During the last 5 years, we performed an average of seven thoracoscopic surgeries for EA/TEF each year. A total of 41 infants, excluding three preterm infants who underwent primary repair with open surgery in neonatal intensive care unit, underwent thoracoscopic repair of EA/TEF between October 2008 and February 2017. For this study, the medical records of these 41 infants were reviewed retrospectively.

The surgical procedure used was the same as that previously described by Lee et al. of this center [10]. The infant was laid in a left semi-lateral decubitus position after general anesthesia. After a 5-mm camera trocar was inserted just below the tip of the scapular, CO_2_ gas was inflated into the thoracic cavity with a flow of 3 L/min, up to a maximum pressure of 5 mmHg. Then, the azygos vein overlying the fistula was divided with electrocautery. After the distal esophagus was identified and dissected from the trachea to the proximal direction, the TEF was ligated by suture ligation with 3–0 braided polyglycolic absorbable sutures (Safil®; B. Braun, Melsungen, Germany). Next, the proximal end was dissected from the adjacent tissue and the pouch was opened for anastomosis. Esophageal anastomosis was done by placing 5–0 glyconate monofilament interrupted sutures (Monosyn®; B. Braun, Melsungen, Germany) around the lumen. Last, a 10-French chest tube was routinely inserted with the tip located near the anastomosis. Following operation, the infant was initially kept on mechanical ventilation for several days. Then, for 7 days, oral intake was restricted with 8-French feeding tube placement. On the seventh postoperative day, barium esophagography was performed. Upon confirming there was no anastomosis leakage or stricture, oral intake was initiated and the chest tube was removed on the following day.

Data on intraoperative events requiring anesthesiologist’s intervention were collected by reviewing the anesthetic records of the patient. Difficult intubation was defined as the need to change the endotracheal tube more than three times during the induction phase of anesthesia. Difficult ventilation was defined by the performance of manual ventilation by the anesthesiologist due to the occurrence of a desaturation event prior to the ligation of the TEF. A hypoxic event was defined as a decrease in pulse oxymeter saturation (SpO_2_) of less than 90% or drop of more than 10% from baseline values.

The intraoperative factors considered herein were hypothermia, maximum EtCO2, pulse pressure variation, and chloride level. Hypothermia was defined as a core temperature of less than 36.0 °C at any point during surgery.

To compare continuous variables, the Mann–Whitney *U* test was used. Conversely, for categorical variable comparisons, the χ^2^ test was employed. *P* values of less than 0.05 were considered to be significant. The Statistical Package for the Social Sciences version 22.0 for Windows software program (IBM Corp., Armonk, NY, USA) was used in all statistical analyses.

This study was approved by the Institutional Review Board at Samsung Medical Center (IRB File No. 2019-05-064-001).

## Results

During the study period, a total of 41 infants underwent thoracoscopic EA/TEF repair at Samsung Medical Center in Seoul, Korea. Patient weights at the time of operation were between 1440 and 4000 g (mean 2604 ± 559 g). The median follow-up period was 43 months (range 1–112 months). One case was converted to open surgery because of a highly located proximal end and the difficulty of dissection between the proximal esophagus and trachea.

Upon subgrouping by weight at the time of operation, seven infants under 2000 g were placed in Group A and 34 weighing more than 2000 g were included in Group B. The demographic characteristics of the infants are listed in Table 1. Group A had more patients with cardiac anomalies (86%) than did Group B (35%) (*P* = 0.031). As shown in Table 2, intraoperative factors and events indicating the safety of surgery and the difficulty of anesthesia were not statistically different between the two groups. Surgical outcomes for both groups are compared in Table 3. Group A required three more days of ventilator support in comparison with Group B (*P* = 0.03). According to postoperative esophagography, no patient (0%) in Group A showed leakage, while 3 (42.9%) patients showed strictures. On the other hand, 5 (14.7%) and 13 (38.2%) patients in Group B, respectively, showed leakage and strictures, but the difference between the two groups was without statistical significance (*P* = 0.57 and 1.00 for leakage and strictures, respectively). In the long-term follow-up, 4 (57.1%) patients in Group A required fundoplication due to severe gastroesophageal reflux (GER), while 6 (17.6%) in Group B underwent the operation for the same reason (*P* = 0.04). To analyze the factors that affect the occurrence of GER requiring fundoplication, additional multivariate analysis was done (Table 4). Various factors such as history of open-heart surgery and anastomosis leakage were significantly associated with fundoplication (*P* = 0.03 and 0.02, respectively).

**Table 1:** Demographic characteristic of the infants

	Overall (*N* = 41)	Group A < 2000 g (*N* = 7)	Group B > 2000 g (*N* = 34)	*P* value
Male:female	24:17	4:3	20:14	1.00
Gestational age (weeks, mean)	37.3	34.1	38.2	0.00
Birth weight (g, mean ± SD)	2612 ± 638	1706 ± 126	2799 ± 529	0.00
Age at operation (days, median)	6 (1–26)	5 (1–26)	7 (1–13)	0.47
Weight at operation (g, mean ± SD)	2604 ± 559	1874 ± 412	2759 ± 457	0.00
Associated anomaly (%)	25 (61)	6 (86)	19 (56)	
Cardiovascular anomaly (%)	18 (44)	6 (86)	12 (35)	0.031
1 major anomalies (%)	7 (17)	1 (14)	6 (18)	1.00

*SD* standard deviation

**Table 2:** Intraoperative outcomes

	Overall (*N* = 41)	Group A < 2000 g (*N* = 7)	Group B > 2000 g (*N* = 34)	*P* value
Operation time (min, mean ± SD)	153 ± 70	140 ± 53	156 ± 75	0.52
Conversion to open surgery (%)	1 (2)	0 (0)	1 (3)	1.00
Intraoperative events and factors				
Difficult intubation (%)	3 (7)	2 (29)	1 (3)	0.07
Difficult ventilation (%)	5 (12)	1 (14)	4 (12)	0.62
Hypoxic event %)	19 (46)	4 (57)	15 (44)	0.41
Hypothermia (%)	11 (27)	4 (57)	7 (21)	0.07
Maximum EtCO2 (mmHg, mean ± SD)	45.98 ± 10.8	42.1 ± 8.7	46.7 ± 11.1	0.31
Pulse pressure variation (mean)	17.2 ± 6.9	12.5 ± 10.6	18.6 ± 5.99	0.30
Chloride (mmol/L, mean ± SD)	111.6 ± 5.4	108.9 ± 6.6	112.2 ± 5.0	0.14
Perioperative body weight change (g, mean ± SD)	163 ± 128.6	121 ± 130.6	172.4 ± 128.4	0.34
RBC transfusion (%)	2 (5)	1 (14)	1 (3)	0.23
Intraoperative vasoconstrictor use (%)	16 (39)	2 (29)	14 (41)	0.66

*RBC* red blood cell, *SD* standard deviation

**Table 3:** Postoperative outcomes

	Overall (N = 41)	Group A < 2000 g (N = 7)	Group B > 2000 g (N = 34)	P value
Mechanical ventilation (days, mean ± SD)	3.9 ± 3.9	6.7 ± 5.2	3.3 ± 3.4	0.03
Full oral feeding (days, mean ± SD)	15.3 ± 7.0	17.2 ± 5.3	14.8 ± 7.3	0.41
Hospital stay (days, mean ± SD)	37.9 ± 54	86.2 ± 99.3	27.9 ± 35.2	0.01
Anastomosis leakage (%)	5 (12)	0 (0)	5 (15)	0.57
Strictures requiring balloon dilatation (%)	16 (39)	3 (43)	13 (38)	1.00
Gastroesophageal reflux (GER)	13 (32)	4 (57)	9 (26)	0.18
Fundoplication (%)	10 (24)	4 (57)	6 (18)	0.04
Mortality (%)	1 (2)	1 (14)	0 (0)	0.171

*SD* standard deviation

**Table 4:** Associations between clinical factors and GER requiring fundoplication

Variables	GER requiring fundoplication (%)			Multivariate analysis	
	No	Yes	*P* value	Adjusted OR (95% CI)	*P* value
Total no	31 (76)	10 (24)			
Weight at operation					
< 2000 g	3 (43)	4 (57)	0.04	10.22 (1.15–90.29)	0.03
> 2000 g	28 (82)	6 (18)			
Heart surgery					
No	30 (83)	6 (17)	0.01	19.64 (1.32–290.66)	0.03
Yes	1 (20)	4 (80)			
Anastomosis leakage					
No	29 (81)	7 (19)	0.04	14.44 (1.33–156.33)	0.02
Yes	2 (40)	3 (60)			

*GER* gastroesophageal reflux, *OR* odd ratio, *CI* confidence interval

## Discussion

Thoracoscopic repair of EA/TEF is technically challenging and remains an unfamiliar procedure for many pediatric surgeons, who mainly deal with intra-abdominal organs. Also, most of the infants who undergo repair for EA/TEF are very small at the time of surgery; the surgical field is small and narrow, making surgeons experience much technical difficulties. Furthermore, in order to do thoracoscopic surgeries, operative pneumothorax is made and the ipsilateral lung is compressed, raising the complexity of anesthetic management eventually [9]. In these circumstances, concerns about treating infants under 2000 g with thoracoscopic procedure have led to a scarcity of both single cases and large-scale trials. Moreover, selection criteria, suggested by Dingermann et al. [9], made surgeons hesitate to operate such small infants by thoracoscopic procedure.

Since the first case of thoracoscopic repair of EA/TEF at our center was performed in October 2008, the number of thoracoscopic cases had gradually increased over the years. In our center, seven infants were under 2000 g; 18 demonstrated accompanying cardiovascular (e.g., atrial and/or ventricular septal defects or reflux) anomalies, and seven had more than one major associated anomaly such as a spinal, limb, renal, and anorectal anomaly. To analyze the safety of the surgery and the difficulty of anesthesia, various intraoperative factors that could influence the surgical outcome were reviewed. Hypercarbia (EtCO2) and hypothermia cause acidosis, which can result in a poor outcome. Pulse pressure variation predicts fluid responsiveness and cardiac function. High chloride may imply an inflammatory status [11, 12]; thus, infants with high perioperative chloride levels might be more at risk of postoperative complications. Upon comparing and analyzing these intraoperative factors according to infant weight, we found that all of the parameters were not significantly different between the two groups (Table 2). Previous studies have suggested difficulties in the anesthetic management of EA/TEF, especially in infants with cardiac anomalies and low birth weights [13, 14]. Upon our review, the infants in Group A, who weighed under 2000 g, had significantly more cardiac anomalies (Table 1). However, despite the higher incidence of cardiac malformations in the Group A, there were no differences in either the intraoperative factors or events between the two groups. We believe that if an advanced anesthetic technique and management are applied, it may be possible to safely perform thoracoscopic surgery in infants weighing less than 2000 g.

Furthermore, most of the postoperative surgical outcomes presented no difference between the two groups, including the rate of postoperative leakage and strictures requiring balloon dilatation (Table 3). In our previous report [10], we discovered that later thoracoscopic cases had significantly shorter operation times and reduced stricture rates. These results provided evidence that a significant learning curve exists for this surgical procedure, and such could definitively affect the surgical outcomes. The difference in the ventilator care period might be caused by the smaller gestational age of the low-birth-weight group, which may imply greater pulmonary immaturity and thus a higher dependence on mechanical ventilation. Separately, the incidence of GER requiring fundoplication was higher in Group A (*P* = 0.04). We analyzed other clinical factors that could possibly occur with GER (Table 4). There were five patients who underwent open-heart surgery and four required fundoplication (80%); meanwhile, of the other 36 patients, 6 (16.7%) underwent fundoplication surgery (*P* = 0.011). Of the five patients who showed anastomotic leakage during esophagography, 3 (60%) required fundoplication, while of the 36 patients without leakage, 7 (19%) required the same (*P* = 0.04). In multivariate analysis, all of these factors were significantly associated with fundoplication. These data suggest that various confounding factors that affect the incidence of GER exist, so additional analysis with more cases is required.

We found an overall mortality rate of 2% (one death in 41 cases). Dingemann et al. [15] suggested meticulous selection criteria for patients, including birth weight over 2000 g, to achieve better outcomes of thoracoscopic repair of EA/TEF and possibly lower the mortality rate. Previous studies have reported their cases of mortality usually occurred in the early postoperative period [16, 17]. However, our center’s single mortality case occurred at 305 days after the repair of EA/TEF, making it difficult to correlate the cause of death with an effect of surgery. In our opinion, low birth weight itself is not a significant factor that could affect mortality in the thoracoscopic repair of EA/TEF.

There are several limitations to this study. First, it was a retrospective study, and three infants underwent open surgery during the study period. Of the three, two of them were under 2000 g, possibly resulting in selection bias. Second, the number of patients included was not large, making it difficult to generalize the present results. However, to our knowledge, this is the first study to compare the outcomes of patients with EA/TEF based on weight at the time of surgery. Hopefully, our findings will serve to help extend the patient selection criteria, leading to more infants with low birth weights receiving minimally invasive surgery in the future. However, for this, more case collection and re-analysis might be required.

In conclusion, the intraoperative and postoperative outcomes of thoracoscopic repair of EA/TEF were similar between the infants who weighed under 2000 g and the ones who were over 2000 g. Therefore, thoracoscopic repair might be a feasible surgical option for infants weighing less than 2000 g when performed by a surgeon and anesthesiologist team who are experienced in pediatric thoracoscopic surgery.
